# Advancement of door-to-needle times in acute stroke treatment after repetitive process analysis: never give up!

**DOI:** 10.1177/17562864221122491

**Published:** 2022-09-15

**Authors:** Johanna Ernst, Kai F. Storch, Anh Thu Tran, Maria M. Gabriel, Andrei Leotescu, Anna-Lena Boeck, Meret K. Huber, Omar Abu-Fares, Paul Bronzlik, Friedrich Götz, Hans Worthmann, Ramona Schuppner, Gerrit M. Grosse, Karin Weissenborn

**Affiliations:** Department of Neurology, Hannover Medical School, Carl-Neuberg Strasse 1, Hannover 30625, Lower Saxony, Germany; Department of Neurology, Hannover Medical School, Hannover, Germany; Department of Anesthesiology, Hannover Medical School, Hannover, Germany; Department of Neurology, Hannover Medical School, Hannover, Germany; Department of Neurology, Hannover Medical School, Hannover, Germany; Department of Neurology, Hannover Medical School, Hannover, Germany; Department of Neurology, Hannover Medical School, Hannover, Germany; Department of Neurology, Hannover Medical School, Hannover, Germany; Department of Neuroradiology, Hannover Medical School, Hannover, Germany; Department of Neuroradiology, Hannover Medical School, Hannover, Germany; Department of Neuroradiology, Hannover Medical School, Hannover, Germany; Department of Neurology, Hannover Medical School, Hannover, Germany; Department of Neurology, Hannover Medical School, Hannover, Germany; Department of Neurology, Hannover Medical School, Hannover, Germany; Department of Neurology, Hannover Medical School, Hannover, Germany

**Keywords:** acute ischemic stroke, delaying factors, door-to-image time, door-to-needle time, image-to-needle time, intravenous thrombolysis, process analysis, process time, sex differences, time is brain

## Abstract

**Background:**

In acute ischemic stroke, timely treatment is of utmost relevance. Identification of delaying factors and knowledge about challenges concerning hospital structures are crucial for continuous improvement of process times in stroke care.

**Objective:**

In this study, we report on our experience in optimizing the door-to-needle time (DNT) at our tertiary care center by continuous quality improvement.

**Methods:**

Five hundred forty patients with acute ischemic stroke receiving intravenous thrombolysis (IVT) at Hannover Medical School were consecutively analyzed in two phases. In study phase I, including 292 patients, process times and delaying factors were collected prospectively from May 2015 until September 2017. In study phase II, process times of 248 patients were obtained from January 2019 until February 2021. In each study phase, a new clinical standard operation procedure (SOP) was implemented, considering previously identified delaying factors. Pre- and post-SOP treatment times and delaying factors were analyzed to evaluate the new protocols.

**Results:**

In study phase I, SOP I reduced the median DNT by 15 min. The probability to receive treatment within 30 min after admission increased by factor 5.35 [95% confidence interval (CI): 2.46–11.66]. Further development of the SOP with implementation of a mobile thrombolysis kit led to a further decrease of DNT by 5 min in median in study phase II. The median DNT was 29 (25th–75th percentiles: 18–44) min, and the probability to undergo IVT within 15 min after admission increased by factor 4.2 (95% CI: 1.63–10.83) compared with study phase I.

**Conclusion:**

Continuous process analysis and subsequent development of targeted workflow adjustments led to a substantial improvement of DNT. These results illustrate that with appropriate vigilance, there is constantly an opportunity for improvement in stroke care.

## Introduction

The slogan ‘time is brain’ dominates acute stroke therapy. In patients suffering acute stroke due to large vessel occlusion, a loss of 1.9 million neurons per untreated minute is estimated.^1^ Favorable clinical outcome after ischemic stroke significantly depends on the timely administration of acute therapies, that is, intravenous thrombolysis (IVT) by recombinant tissue-type plasminogen activator (rt-PA) and mechanical thrombectomy.^2,3^ Thus, every effort should be made to keep the time interval between hospital admission and administration of rt-PA (door-to-needle time [DNT]) as short as possible.

The DNT may be divided into two intervals: The interval from admission to primary cerebral imaging (door-to-image time [DIT]) and the interval between imaging and start of treatment with rt-PA (image-to-needle time [INT]).^4^

A multitude of different reasons affect and may delay workflow, including patient-related factors like uncontrolled hypertension, agitation or vomiting, and also shortcomings in process organization, such as missing pre-notification by emergency medical services (EMS) or delay in brain imaging.^5^ Some factors only affect the DIT, for example, a crowded emergency room (ER) or the scanner localization.^6,7^ In particular, fluctuations in INT, which have a variety of causes, are responsible for the variability of DNT.^4^

Since the introduction of IVT, neurologists have attempted to reduce DNT to improve patients’ outcome.^8–15^ With CODE STROKE, first established in 1994, neurologists initiated new structures in acute stroke treatment, for example, by introducing a single-call activation as well as monitoring of treatment times.^12^ Further development of this protocol resulted in EMS pre-notification, reservation of computed tomography (CT)–scanner and administering rt-PA in the imaging area.^14^ In 2017, Kamal *et al.*^13^ showed that a rapid patient registration, direct referral to the CT imaging and administration of rt-PA at the scanner area had significant impact upon DNT. To summarize, a variety of different improvement strategies have been proposed which on their own or in concert can significantly reduce stroke treatment times.^11^ Aiming at an effective improvement of the DNT at our center, we decided to prospectively analyze the workflow between arrival of patients with acute ischemic stroke considered in need for IVT and start of rt-PA application. Thereby, nine possibly delaying factors were identified, which were addressed in a new standard operation procedure (SOP) I, which was prospectively evaluated thereafter. In a second step, the long-term effect of SOP I and the effect of an amendment to the SOP (i.e. SOP II) were retrospectively assessed.

## Methods

The corresponding author had full access to all the data in the study and takes responsibility for its integrity and the data analysis. Raw data supporting the findings of this study are available upon reasonable request.

### Study population

**Figure 1.**
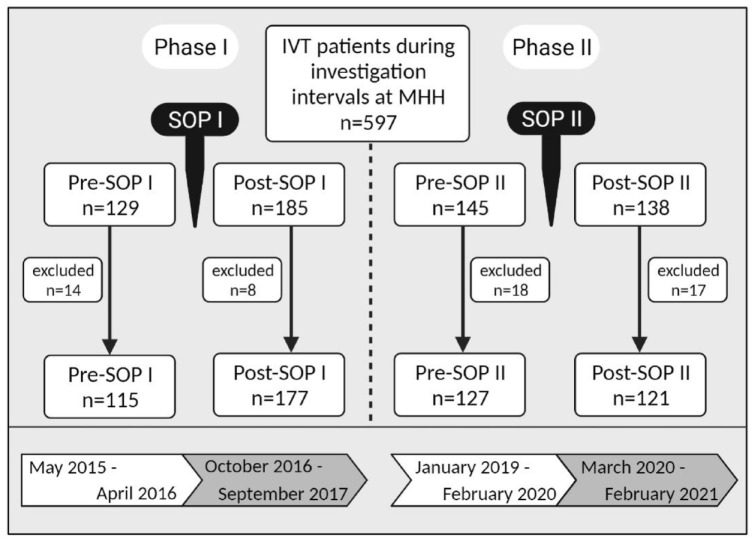
Overview of study population: Data of 597 patients have been collected. Phase I: pre- and post-SOP I: 314 patients received an IVT of whom 22 patients have been excluded. Phase II: pre- and post-SOP II: 283 patients receiving an IVT of whom 35 patients have been excluded. IVT, intravenous thrombolysis; MHH, Hannover Medical School; *n*: number of patients.

In the present study, we performed an internal quality control of acute ischemic stroke therapy in two subsequent phases. In phase I, 1684 patients, and in phase II, 1777 patients with acute ischemic stroke were admitted to Hannover Medical School. Each of the two phases consisted of two intervals – pre- and post-implementation of a new SOP – for which patients were consecutively analyzed. This resulted in a workflow analysis of in total 597 patients with acute ischemic stroke who received IVT. Of these 597 patients, 57 were finally excluded due to exclusion criteria (Figure 1). Exclusion criteria are specified in Table 1.

**Table 1.** Exclusion criteria and excluded patients in study phases I and II.

Exclusions phase I (*n*)	Exclusion criteria	Exclusions phase II (*n*)
7	Intubation before cerebral imaging	10
4	Stroke after hospitalization	4
7	Secondary onset/aggravation of symptoms after hospitalization	4
2	IVT decision was not made by the ER physician	0
0	Retinal artery occlusion	9
2	Missing/incomplete documentation	8

ER, emergency room; IVT, intravenous thrombolysis.

### Study procedures

#### Phase I

From May 2015 until April 2016 (pre-SOP I), data from 115 consecutive patients with acute ischemic stroke receiving IVT were collected prospectively. The following parameters were recorded: National Institutes of Health Stroke Scale (NIHSS), localization of infarction, age, sex, family and insurance status of the patient, weekday of treatment, admittance during regular working hours or during on-call service, mode of referral, ER structure (number of neurologists working in the ER, number of neurological patients referred to the ER within ± 1 h of the referral of a stroke patient, and seniority of the ER neurologist in charge), pre-notification by the EMS, time of symptom onset, modality of imaging (CT or magnetic resonance imaging [MRI]), DIT, INT, DNT, and distinct reasons for delay (e.g. hypertensive crisis, vomiting, waiting time until diagnostic imaging was available). Noteworthy, according to an internal protocol, we regularly performed an MRI in case of an unknown or extended time of onset.^16^

After completion of the respective data analysis in 2016 and interdisciplinary discussion of the results in accordance with the current status of literature,^8,9,14,17–19^ SOP I was elaborated in cooperation with all players involved in the process of acute stroke treatment, that is, neurologists and nurses at the ER and the stroke unit (SU) as well as neuroradiologists. SOP I was valid in October 2016. All measures taken in SOP I are summarized in Table 2.

**Table 2.** Similarities and differences of SOP I and SOP II..

	A	B
	SOP I	SOP II
**1**	Advance notification by the EMS, that a patient with presumed stroke within the time window for IVT is being transferred to the ER	
**2**	Neurologist und ER nurse stay in the ER to meet the patient immediately after admission	
**3**	Pre-emptory registration of an emergency CCT and blood examinations after pre-notification	
**4**	CCT is reserved for the stroke patient after pre-notification	+SU nurse with thrombolysis kit is informed to meet the neurologist at the scanner area
**5**	Emergency transport within the hospital by the neurologist on duty	
**6**	Reporting radiologist is present at the CT room while examination is performed	+SU nurse with thrombolysis kit is present at the CT room while examination is performed
**7**	Decision for or against IVT is made immediately after the NCCT has been finished and before starting CTA	
**8**	Ordering of rt-PA bolus and infusion at the stroke unit after NCCT has been evaluated	Preparation of rt-PA bolus at the CT room and immediate start of rt-PA bolus injection in the CT scanner before CTA is running
**9**	rt-PA is prepared and brought to the CT room while CTA is running	Preparation of rt-PA infusion in the scanner area while CTA is running
**10**	Immediate start of rt-PA bolus injection in the CT scanner after CTA was performed, and transport to the stroke unit with dripping rt-PA infusion	Immediate start of rt-PA infusion in the CT scanner after CTA was performed and transport to the SU with dripping rt-PA infusion
**11**	General rules: Treatment decision is made as an emergency decision without consultation of absent relatives and without waiting for laboratory results	

EMS, emergency medical service; IVT, intravenous thrombolysis; ER, emergency room; CCT, cranial computed tomography; SU, stroke Unit; rt-PA, recombinant tissue plasminogen activator; CTA, computed tomography with angiography.

SOP I implemented after April 2016 combined 11 important items to improve DNT. SOP II implemented in March 2020 introduced the mobile thrombolysis kit for administering rt-PA in imaging area.

From October 2016 until September 2017 (post-SOP I), process times were prospectively recorded again including 177 patients receiving IVT for acute stroke therapy.

#### Phase II

To assess the sustainability of SOP I, DNTs of another 127 patients, who had received IVT for acute ischemic stroke between January 2019 and February 2020, were evaluated retrospectively (pre-SOP II). After this, a mobile thrombolysis kit was implemented in accordance with Tahtali *et al.*^15^ as a part of an adjusted SOP (SOP II). SOP II was valid in March 2020 and included advance notice of an incoming stroke patient with probable indication for IVT to an SU nurse, presence of an SU nurse with a mobile thrombolysis kit at the scanner area according to Tahtali *et al.*^15^ and administration of the rt-PA bolus immediately after completion of the noncontrast cerebral computed tomography (NCCT) (Table 2).

The effect of SOP II was assessed by another retrospective analysis of DNT, which included 121 consecutive patients who were treated between March 2020 and February 2021 (post-SOP II).

##### Overall information for SOP I and II

Patients who arrived within a 4.5-h time window received an NCCT. The protocol included an NCCT and a CT-angiography (CTA). For patients admitted in an unclear time window, an MRI was indicated to look for a mismatch between diffusion-weighted imaging (DWI) and fluid-attenuated inversion recovery (FLAIR) imaging. The MRI protocol used includes following sequences: FLAIR axial, DWI axial and coronal, time-of-flight angiogram and an axial susceptibility-weighted imaging. If a brainstem infarction was suspected a sagittal DWI was added.

The time point, at which the CTA was started, differed between SOPs I and II (Table 2): patients in phase I received IVT after NCCT and CTA had been finished. In phase II, patients were treated with IVT directly after NCCT before CTA was running.

In general, in every phase of the study, rt-PA was started without waiting for coagulation test results or blood cell count. Even if a patient was known to be treated with a vitamin K antagonist, IVT was started without waiting for the International Normalized Ratio (INR) result. However, IVT was stopped immediately if the INR was higher than 1.7. We were able to do so, because blood samples were sent to the laboratory with the highest priority, so that anticoagulation results and blood cell count were available within 30 min after admission.

To evaluate the correct dosage for IVT, patients’ weight was estimated by the ER neurologist.

#### Data acquisition

We extracted relevant data from computerized clinical documentation systems, picture archiving and communication system, EMS protocols, patients’ case records and clinical protocols. For the prospective analysis, interviews with treating physicians within 24–48 h after IVT were performed, to inquire about perceived causes of DNT delay, such as slow workflow or waiting time at the ER or imaging center, lack of an intravenous (IV) catheter, unknown time of symptom onset, assertion of indications or contraindications by detailed interview of patient or relatives, uncontrolled hypertension, uncertainty about diagnosis, agitation, or vomiting needing treatment before further diagnostics or unexplainable delay of thrombolysis after delivery of the imaging results.

#### Statistical analysis

Statistical analysis was done using IBM SPSS Statistics 27 and SAS Enterprise Guide 7.1. Numbers and percentages were used to describe categorical variables and median and 25th–75th percentiles for non-normally distributed continuous variables. Group comparisons were done using the Mann–Whitney *U*-test and Kruskal–Wallis for non-normally distributed continuous data and the chi-square test or Fisher’s exact test, as appropriate, for comparison of categorical data. For categorical data relative risks (RRs) with 95% confidence intervals (CIs) were calculated, adjustment for potential confounders was done using the Cochran–Mantel–Haenszel test. A linear regression model was established in the prospective cohort including DNT as dependent variable and all factors as independent variables that were regarded as potential confounders according to the baseline analysis (see Supplement Tables 1 and 2). Figures were created using biorender.com and GraphPad Prism 9.0.1. Boxplots with Tukey whiskers are shown unless reported otherwise.

## Results

### Study population

Clinical characteristics of the study groups are summarized in Table 3. No relevant differences were observed regarding age, sex, stroke severity, primary image modality, anticoagulation status, and localization of infarction. The prevalence of an unknown time window was higher in post-SOP I (35.6%) than in pre-SOP I (20.9%), whereas no difference in distribution of an unclear time window in pre- and post-SOP II (pre-SOP I: 28.6% *versus* post-SOP I: 29.8%) was detectable. The rates of IVT-treated patients did not differ substantially between the four study intervals, whereas in post-SOP I, a higher percentage of IVT-treated patients was recognized (pre-SOP I: 15.5% *versus* post-SOP I: 21.7% *versus* pre-SOP II: 15.9% *versus* post-SOP II: 15.9%). We could not find a clear explanation for the increased IVT rate in post-SOP I. Table 4 contains the prevalence and distribution of potential DNT delaying factors in the prospective phase I cohort.

**Table 3.** Baseline characteristics.

Baseline characteristics	Pre-SOP I	Post-SOP I	Pre- SOP II	Post-SOP II	*p* value
	*n* = 115	*n* = 177	*n* = 127	*n* = 121	
Female, *n* (%)	44 (38.3)	95 (53.7)	59 (46.5)	63 (52.1)	0.056
Age, years (25th–75th pct)	75 (65–84)	77 (66–84)	78 (63–85)	75 (61.5–83)	0.734
NIHSS (25th–75th pct)	7 (4–11)	8 (4–13)	7 (4–13)	7 (4–13)	0.462
Patients on anticoagulation, *n* (%)	3 (2.6)	2 (1.1)	4 (3.1)	4 (3.3)	0.580
Unknown time of onset, *n* (%)	24 (20.9)	63 (35.6)	36 (28.6)	36 (29.8)	0.062
Onset to door time,^a^ minute (25th–75th pct)	69 (47–120)	60 (43–90.25)	70 (49–109)	76 (55–121)	0.034
Anterior cerebral circulation, *n* (%)	101 (87.8)	153 (86.4)	102 (80.3)	105 (86.7)	0.320
Primary cCT, *n* (%)	90 (78.3)	140 (79.1)	97 (76.4)	89 (73.6)	0.710
Median DNT, minute (25th–75th pct)	51 (40–64)	36 (30–46)	34 (25–48)	29 (18–44)	< 0.001

CT, computed tomography; DNT, door-to-needle time; NIHSS, National Institutes of Health Stroke Scale; pct, percentile; SOP, standard operation procedure.

a In case of a clear onset.

**Table 4.** Prevalence of potential DNT delaying factors in the prospective cohort.

		Pre-SOP I, *n* = 115 (%)	Post-SOP I, *n* = 177 (%)	*p* value		
Insurance status	PHI	29 (25.2)	47 (26.6)	0.799	Private conditions	
	SHI	86 (74.8)	130 (73.4)			
Family condition	Single	15 (13.0)	39 (22.0)	0.113		
	Partnership	67 (58.3)	99 (55.9)			
	Unclear	33 (28.7)	39 (22.0)			
Residential condition	At home	100 (87.0)	139 (78.5)	0.247		
	Nursing home	11 (9.6)	32 (18.1)			
	Other	4 (3.5)	6 (3.4)			
Mode of referral	EMS + EP	35 (30.4)	38 (21.5)	0.268	Prehospital conditions	
	EMS	72 (62.6)	127 (71.8)			
	Private	3 (2.6)	7 (4.0)			
	Unclear	5 (4.3)	5 (2.8)			
Pre-notification	Yes	90 (78.3)	145 (81.9)	0.553		
	No	21 (18.3)	29 (16.4)			
	Unclear	4 (3.5)	3 (1.7)			
Timepoint of admittance	Weekday	84 (73.0)	134 (75.7)	0.609	Time	
	Weekend	31 (27.0)	43 (24.3)			
	Working hours	51 (44.3)	70 (39.5)	0.416		
	On-call hours	64 (55.7)	107 (60.5)			
No. of neurologists working in the ER	1	29 (25.2)	80 (45.2)	0.001	Emergency room structure	
	2	86 (74.8)	97 (54.8)			
No. of neurological patients referred to ER within ± 1 h of the referral of a stroke patient	0	44 (38.3)	48 (27.1)	0.037		
	1–3	64 (55.7)	105 (59.3)			
	4–7	7 (6.1)	24 (13.6)			
Work experience of ER neurologist in charge in years	1–3	74 (64.3)	94 (53.1)	< 0.001		
	4–5	23 (20.0)	50 (28.2)			
	6–7	18 (15.7)	33 (18.6)			
Acute treatment of elevated blood pressureAgitation and vomiting		24 (20.9)	32 (18.1)	0.554	Patient-related factors	Further observed reasons for delay
		15 (13.0)	12 (6.9)	0.071		
Consultation of relatives		6 (5.2)	1 (0.6)	0.016		
Arrival without IV catheter		12 (10.4)	25 (14.1)	0.354	System-related factors	
Waiting for brain imaging		24 (20.9)	28 (15.8)	0.270		
ER treatment > 10 min		5 (4.3)	20 (11.3)	0.052		
Delay with unclear reason		12 (10.4)	2 (1.1)	< 0.001		
Indication for IVT is disputable		25 (21.7)	32 (18.1)	0.441		
Technical difficulties with rt-PA		3 (2.6)	2 (1.1)	0.386		

EMS, emergency medical service; EP, emergency physician; ER, emergency room; IV, intravenous; IVT intravenous thrombolysis; PHI, private health insurance; rt-PA, recombinant tissue type plasminogen activator; SHI, statutory health insurance.

### Role of delaying factors

In study phase I, the effect of all factors mentioned in Tables 3 and 4 on median DNT was calculated. The median DNT showed a broad range depending on presence or absence of the various possibly delaying factors. Although the evidence for differences was modest for most factors in the pre-SOP I period due to small sample sizes they were considered as clinically relevant. This assumption is supported by the data from the post-SOP I period with considerably larger sample size, where statistical significance was achieved for most of them in a univariate analysis (Supplement Tables 1 and 2).

**Figure 2.**
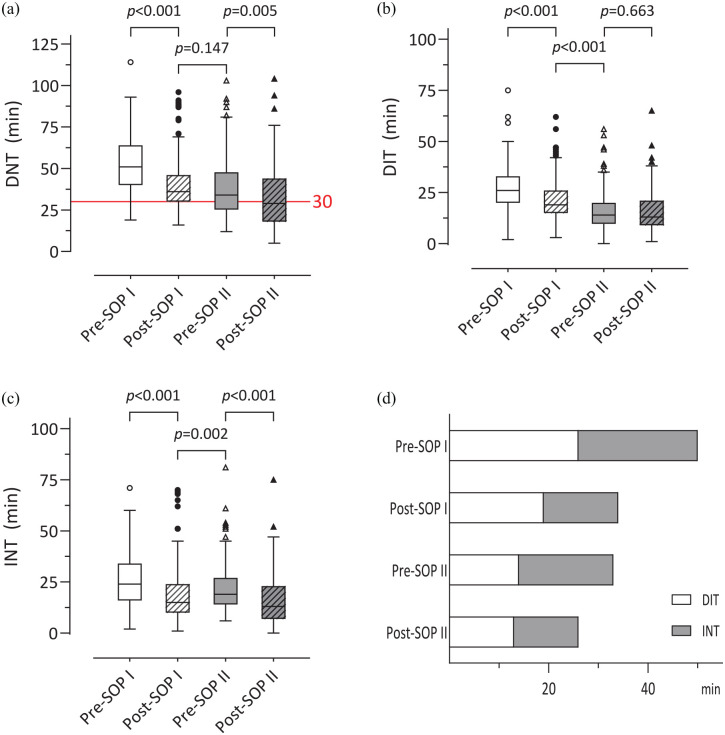
Timeline of DNT, DIT and INT during study phases: (a) DNT decreased after SOPs I and II. There was no difference between post-SOP I and pre-SOP II. (b) DIT decreased after SOP I. No difference of DIT was found between pre- and post-SOP II. (c) INT decreased after SOP I as well as after SOP II but slightly increased between post- and pre-SOP II. (d): Median DNT were reduced by SOPs I and II. Boxplots with Tukey whiskers are shown where applicable. DIT, door-to-imaging time; DNT, door-to-needle time; INT, imaging-to-needle time.

Considering the data from pre-SOP I analysis, all stakeholders involved in acute stroke treatment as described above agreed upon the following measures to improve DNT (SOP I): pre-notification of an acute stroke patient to the ER neurologist by the EMS, effective reservation of the CT for the stroke patient, presence of the reporting radiologist at the CT, decision-making for or against IVT immediately after NCCT, ordering rt-PA immediately after decision-making and administration of rt-PA in the scanner area after CTA was running (Figure 2(a)).

SOP I significantly reduced the prevalence of delaying factors; 9.6% of patients in pre-SOP I and 31.1% in post-SOP I had no documented delaying factors (*p* < 0.001) (see Supplemental material). Furthermore, consultation of relatives (pre-/post-SOP I, 5.2% *versus* 0.6%, *p* = 0.016) and unclear delays (10.4% *versus* 1.1%, *p* < 0.001) were reduced, whereas a higher percentage of ER treatment over 10 min without another clear reason of delay (pre-/post-SOP I, 4.3% *versus* 11.3%, *p* = 0.052) was observed (Table 4).

A multiple linear regression analysis including baseline characteristics as well as those prospectively gathered parameters that had shown significant impact upon DNT in univariate group comparisons (Supplement Table 1) in post-SOP I identified age and imaging modality, the number of neurologists working in the ER and most of the further observed reasons for delay as independent DNT-influencing factors (Table 5). Of note, women received treatment in 55 (25th–75th percentiles: 43.25–71.5) min compared with 46 (25th–75th percentiles: 36–60) min for men in pre-SOP I (see Supplemental material). In both periods, women were significantly older than men [median age women *versus* men, pre-SOP I: 81 (25th–75th percentiles: 65.75–86) years *versus* 73 (25th–75th percentiles: 61–81) years, *p* = 0.012, post-SOP I: 80 (25th–75th percentiles: 74–87) years *versus* 73 (25th–75th percentiles: 60–80) years, *p* < 0.001]. According to the linear regression analysis, sex was not independently associated with DNT, and thus, longer treatment times of women likely were due to confounding.

**Table 5.** Linear regression model with impact of potential delaying factors on DNT.

		Pre-SOP I		Post-SOP I	
		β	95% CI	β	95% CI
Sex	Male (ref.)	6.69	–0.32 to 13.70	2.80	–0.96 to 6.55
	Female				
Age	< 75 (ref.)	3.73	–2.78 to 10.23	4.55	0.49–8.61
	⩾ 75				
NIHSS	0–4	–0.11	–6.98 to 6.76	–0.57	–3.54 to 2.40
	5–15				
	16–42				
Clear time of onset (ref.)		2.77	–6.60 to 12.14	–1.46	–5.61 to 2.69
Unclear time of onset					
Anterior cerebral circulation (ref.)		8.08	–1.83 to 17.99	3.04	–2.14 to 8.22
Posterior cerebral circulation					
Cranial computed tomography (ref.)		13.37	3.81–22.94	6.81	1.74–11.88
Cranial magnetic resonance imaging					
Pre-notification	Yes	0.157	–6.59 to 6.91	3.05	–1.13 to 7.22
	No				
	Unclear				
No. of neurologists working in the ER	1 (ref.)	–0.30	–8.28 to 7.69	–4.32	–7.94 to 0.70
	2				
Work experience of ER neurologist in years	1–3	1.43	–2.89 to 5.75	1.16	–1.16 to 3.48
	4–5				
	6–7				
Acute treatment of elevated blood pressure		1.84	−6.49 to 10.17	5.65	0.90–10.39
Agitation and vomiting		3.37	−6.26 to 13.00	24.65	17.52–31.79
Arrival without IV catheter		4.23	−6.21 to 14.67	1.87	−3.34 to 7.08
Waiting for brain imaging		2.09	−5.72 to 9.89	12.14	7.26–17.03
ER treatment over 10 min without another clear reason of delay		−1.61	−15.9 to 18.62	15.23	9.59–20.87
Indication for IVT is not clear		3.71	−4.41 to 11.83	13.53	8.81–18.26

ER, emergency room; IV, intravenous; IVT, intravenous thrombolysis; NIHSS, National Institutes of Health Stroke Scale; No., number; ref., reference.

Influence of baseline characteristics and further observed reasons for DNT delay. In a linear regression model, using data from post-SOP II age, imaging modality, numbers of neurologists in the ER and all observed reasons for delay with exception of missing IV catheter reached statistical significance as an independent factor for DNT delay.

### In-hospital process times (DNT, DIT, and INT) were reduced by SOPs I and II

Patients in pre-SOP I received IVT in a median DNT of 51 (25th–75th percentiles: 40–64) min. After implementation of SOP I, DNT was reduced by 15 min in median [51 (25th–75th percentiles: 40–64) min *versus* 36 (25th–75th percentiles: 30–46) min, *p* < 0.001] (Figure 2(a)). In pre-SOP I, seven out of 115 patients received IVT within 30 min from admission, whereas in post-SOP I, 53 out of 177 were treated in a 30-min interval. SOP I thus led to a nearly by a factor of five increased probability of a DNT below 30 min (RR: 4.92, 95% CI: 2.32–10.44), although in post-SOP I, a higher percentage of patients came in with unknown time of stroke onset which was associated with a longer DNT [58 (25th–75th percentiles: 45.5–67.25) min *versus* 48 (25th–75th percentiles: 38–63) min, *p* = 0.035] (see Supplemental material), likely due to longer imaging protocols. According to adjustment via Cochran–Mantel–Haenszel test for an unknown stroke onset, the probability of a DNT below 30 min was estimated as being 5.35 times as high as in post-SOP I than in pre-SOP I (RR: 5.35, 95% CI: 2.46–11.66).

Median DNT remained constant between post- and pre-SOP II [36 (25th–75th percentiles: 30–46) min *versus* 34 (25th–75th percentiles: 25–48) min, *p* = 0.169]. After SOP II came into effect, the DNT was further reduced by about 5 min [pre-SOP II *versus* post-SOP II: 34 (25th–75th percentiles: 25–48) min *versus* 29 (25th–75th percentiles: 18–44) min, *p* = 0.005] (Figure 2(a)). Fifty-one patients out of 127 in pre-SOP II and 65 out of 121 patients in post-SOP II received treatment within 30 min. The probability of an IVT within 30 min in post-SOP II increased by factor 1.36 (RR: 1.36, 95% CI: 1.04–1.78) compared with pre-SOP II. A DNT of 15 min was achieved in five patients out of 127 in pre-SOP II and in 20 patients out of 121 in post-SOP II. Thus, SOP II led to by a factor of 4.2 increased probability of treatment within a 15-min interval (RR: 4.2, 95% CI: 1.63–10.83).

DIT was reduced by 7 min comparing pre- and post-SOP I [26 (25th–75th percentiles: 20–33) min *versus* 19 (25th–75th percentiles: 15–26) min, *p* < 0.001]. Although the clinical procedure remained the same on principle, a further diminution of DIT was recognized comparing post-SOP I and pre-SOP II [19 (25th–75th percentiles: 15–26) min *versus* 14 (25th–75th percentiles: 9–20) min, *p* < 0.001]. SOP II did not further influence DIT [14 (25th–75th percentiles: 9–20) min *versus* 13 (25th–75th percentiles: 9–21) min, *p* = 0.663] (Figure 2(b)).

INT decreased comparing pre- and post-SOP I [24 (25th–75th percentiles: 16–34) min *versus* 15 (25th–75th percentiles: 10–24) min, *p* < 0.001] and pre- and post SOP II [19 (25th–75th percentiles: 14–27) min *versus* 13 (25th–75th percentiles: 7–23) min, *p* < 0.001]. INT increased between post-SOP I and pre-SOP II [15 (25th–75th percentiles: 10–24) min *versus* 19 (25th–75th percentiles: 14–27) min, *p* = 0.002] (Figure 2(c)).

See Figure 2(d) for an overview of the changes of DNT between the study groups.

## Discussion

We performed a two-step quality analysis of process times in acute stroke treatment with implementation of two new SOPs which finally led to a DNT reduction of over 20 min resulting in a median DNT of 29 min. With implementation of the first SOP, the probability of receiving treatment within 30 min after admission increased by a factor of 5. With the second SOP, valid the probability to receive IVT within 15 min after admission increased by four times.

In 2015, we started a process analysis of diagnostic and therapeutic pathways in our tertiary stroke care center aiming at an improvement of DNT. Supported by literature analysis nine factors were identified, which could potentially delay IVT in patients with acute ischemic stroke within the diagnostic and therapeutic process.^8,18–21^ These delaying factors were divided into patient-related factors, such like agitation, vomiting or uncontrolled hypertension and system-related factors like waiting for brain imaging or delay in decision-making and start of IVT. Furthermore, we analyzed the influence of the patients’ social condition, ER structure, time of admission, and prehospital factors. A prospective analysis showed that several of these factors led to a clinically relevant prolonged median DNT (see Supplemental material). Noteworthy, an unclear time of onset is an important delaying factor. In this case, an MRI is indicated according to Thomalla *et al.*,^16^ which inevitably leads to longer treatment times compared with diagnostics based on CT. Of note, at our center, we already performed an MRI to evaluate a wake-up stroke patient as an internal protocol since 2014.

Based on this analysis and available literature with emphasis on the Helsinki stroke model,^22^ a new SOP (SOP I) was developed and implemented in 2016 (Table 2). Relevant improvement strategies of SOP I were EMS pre-notification,^23^ pre-emptory patient registration, alarming neuroradiologists and scanner preparation prior to arrival,^8,9^ rapid patient transport to the scanner,^22^ and administration of rt-PA in the scanner area.^24^ Meretoja *et al.*^17^ indicated, that the ‘direct into CT-step’ is one of the most effective strategies to reduce DNT. In our center, we had a structural limitation that consists of a distance of about 150 m between ER and scanner area. Moreover, in our center, EMS in general refers the patients to the ER, exclusively, and leaves after handing over. Hence, we were unable to implement the ‘direct into CT-Step’. In SOP I, rt-PA was prepared at the SU and brought to the scanner area after a treatment decision was made. This is another relevant difference to the Helsinki stroke model.^22^ Nevertheless, SOP I reduced median DNT by 15 min. In addition, a reduction of the prevalence of delaying factors was detectable (Table 4). As anticipated, patients without any workflow delay received IVT in median within 30 min, but with increasing number of potentially delaying factors present DNT steadily increased in every single case (Supplemental Table 3).

In our prospective cohort, several structural- and process-related reasons for delay were associated with a longer DNT in the univariate analysis. Combining these factors as independent variables in a multivariate linear regression model age, imaging modality, number of neurologists in the ER, and all previously observed reasons for delay with exception of missing IV catheter reached statistically significance as an independent factor for DNT delay, thus supporting SOP I (Table 5). Recently, we reported on a workflow analysis for endovascular treatment of ischemic stroke at our center, which, in concordance with the present study, revealed that a steady process analysis and a distinct knowledge of potential delaying factors can improve stroke care. Both analyses at our center showed that stroke treatment times where shorter if the ER was staffed with two neurologists. Obviously, patients’ treatment and management is more sufficient when two neurologists are able to share their tasks in a structured manner or if one neurologist could focus exclusively on stroke care, whereas the other colleague could take care of other patients. SOP I may have had a benefit on task sharing as well, whereas, it was more efficient to share tasks with the help of a structured SOP. Thus, support by another person, for example, a stroke nurse, who could be in charge if a stroke patient is pre-notified by EMS, could be a further improvement, if only one neurologist is working in the ER.

However, in the present analysis, we could not identify an influence of on-call service *versus* business times on DNT in contrast to the analysis of endovascular treatment times where door-to-groin-times were shorter during on-call times,^21^ and also in contrast to Groot *et al.*,^25^ who identified a minimal prolonged DNT during on-call-service which was considered as irrelevant.

Post-SOP II covered the first phase of the coronavirus pandemic. The impact of the pandemic on acute stroke care has received increasing attention. Rinkel *et al.*^26^ and Richter *et al.*^27^ reported constant IVT rates comparing the pre-COVID-19 to the COVID-19 period. Regarding stroke process times the current literature yielded heterogeneous findings: In some studies, a delay of the DNT was recognized,^28,29^ whereas, another study showed a constant DNT comparing the pre-COVID-19 and COVID-19 period.^30^ Our study SOP II was able to reduce DNT, despite a rapidly developing pandemic situation.

Kamal *et al.*^13^ reported mixing and administering rt-PA in the imaging area directly after decision-making as the most important factor to reduce DNT. Before implementation of SOP I, rt-PA was not administered before the patient had been transferred to the SU where an SU nurse waited with rt-PA bolus and infusion. SOP I introduced ordering rt-PA to the scanner room immediately after the evaluation of the NCCT and – in accordance with the Helsinki stroke model – administration of rt-PA in the scanner room after CTA was performed. In 2017, Tahtali *et al.* published a protocol that implemented that an SU nurse brings a thrombolysis kit to the scanner area. The thrombolysis kit contained rt-PA and important emergency medication, for example, IV blood pressure medication, IV sedatives, and IV anti-emetic medication.^15^ Based on their contribution, we developed SOP II, which included (1) the delivery of a thrombolysis kit to the scanner area by an SU nurse as soon as an IVT candidate was announced by the ER neurologist and (2) preparation and administration of the IVT bolus on site directly after NCCT and before CTA had started. The infusion was started directly after CTA was finished. To organize this protocol, the ER neurologist had to call the SU nurse directly after pre-notification by the EMS and after reservation of the CT scanner. This again, led to a significant DNT improvement. We were able to reduce treatment time by further 5 min resulting in a median DNT of 29 min. As expected, SOP II only had an effect on INT. This could be taken as an internal control of the efficacy of SOP II, because it did not address and consecutive not affect DIT. It was crucial to reduce INT because it increased between post-SOP I and pre-SOP II with unexplained fluctuations, and in literature, it is described as a more common contributor to delays in stroke therapy.^4^ With SOP II, we were able to increase the rate of patients being treated within 15 min from 3.9 to 16.5%. Our data suggest that a steady process analysis is worthwhile since it improves the workflow in diagnosis and treatment of patients with acute ischemic stroke, and thereby contributes to improved patient outcomes.^15,31^ The first SOP put emphasis on the collaboration of EMS, ER neurologist, ER nurses and the neuroradiology department. In the second SOP, an upgrade was made by bringing ER neurologist, SU nurse, and a neuroradiologist together to concentrate decision-making and treatment as close as possible. The slogan ‘time is brain’ was complemented by the insight ‘team is brain,’ which was described by Tahtali *et al.*^15^ Further improvement of DNT might be achieved by simulation-based training including all specialists and professions involved in the diagnosis and treatment of acute ischemic stroke. This may lead to further long-term reduction of DNT and is already established in cardiovascular life support.^15^ At our center the next step to minimize DNT could be a ‘single-call activation’ as a CODE STROKE, which is an established tool elsewhere^9,13,17,24,32^ using an app-based stroke alarm system, like Noone *et al.* described. The app includes IVT checklists, real time data, and enables the synchronization of all needed on-call members for acute stroke treatment. It was shown to be able to reduce the median DNT from 57 to 41 min in another center.^33^ Other app-based acute stroke care systems were shown to be very sufficient as well.^34,35^

Our study has several limitations. The first study phase (pre- and post-SOP I) consisted of prospective data, whereas, the second phase was a retrospective analysis. As a result, in study phase II, we had to exclude several patients because of incomplete documentation. Furthermore, reasons for delay were not recorded in study phase II. Thus, we were not able to compare both study phases regarding the impact of different reasons for delay.

In general, an experienced neurologist is able to interpret NCCT and to indicate IVT. In our study, a neuroradiologist was present in both phases during every NCCT in acute stroke therapy, but this is most likely a privilege of a university hospital. Thus, in this point, our procedures will not be generalizable to a majority of different hospitals. This, however, supports the incentive to perform center-based process analyses to further optimize stroke care.

Moreover, the introduction of both SOPs could have had a biased effect on DNT via increased motivation and alertness. Therefore, we postulate that another evaluation is necessary to investigate whether the described effects will be long-term improvements or fade away with time.

In conclusion, our workflow analysis on thrombolysis in acute ischemic stroke treatment covering the time interval from 2015 to 2021 and including the implementation of two new SOPs led to a median DNT reduction of over 20 min. We should come to appreciate that ‘team-is-brain’ is as much a slogan as ‘time is brain’ to find further ways of DNT reduction. Finally, stroke care can be steadily improved with continuous alertness and targeted adaptation of workflows.

## Supplemental Material

sj-docx-1-tan-10.1177_17562864221122491 – Supplemental material for Advancement of door-to-needle times in acute stroke treatment after repetitive process analysis: never give up! Click here for additional data file. Supplemental material, sj-docx-1-tan-10.1177_17562864221122491 for Advancement of door-to-needle times in acute stroke treatment after repetitive process analysis: never give up! by Johanna Ernst, Kai F. Storch, Anh Thu Tran, Maria M. Gabriel, Andrei Leotescu, Anna-Lena Boeck, Meret K. Huber, Omar Abu-Fares, Paul Bronzlik, Friedrich Götz, Hans Worthmann, Ramona Schuppner, Gerrit M. Grosse and Karin Weissenborn in Therapeutic Advances in Neurological Disorders

## Declarations

**Ethics approval and consent to participate:** According to local legislation, informed consent or ethical board approval for this study were not required since the analysis was performed as part of clinical workflow quality improvement measures.

**Consent for publication:** Not applicable.

**Availability of data and materials:** Not applicable.
