# Supplementary material for: Advancement of door-to-needle times in acute stroke treatment after repetitive process analysis: never give up!
Source: Ther Adv Neurol Disord. 2022 Sep 15;15:17562864221122491. doi: 10.1177/17562864221122491 (PMC9486271; doi:10.1177/17562864221122491)
Supplement: sj-docx-1-tan-10.1177_17562864221122491 – Supplemental material for Advancement of door-to-needle times in acute stroke treatment after repetitive process analysis: never give up! [file sj-docx-1-tan-10.1177_17562864221122491.docx]

**Online Supplement**

**Supplemental Table 1:** Impact of DNT influencing factors in a univariate analysis in the prospective cohort

|  | | Pre-SOP I | | Post-SOP I | |
| --- | --- | --- | --- | --- | --- |
|  |  | DNT (median in min (25^th^ – 75^th^ percentile)) | p - value | DNT (median in min (25^th^ – 75^th^ percentile) | p - value |
|  |  |  |  |  |  |
| Insurance status | PHI | 44 (36.5-60) | 0.144 | 37 (32-46) | 0.241 |
|  | SHI | 53 (40-65) |  | 35 (28.75-46) |  |
| Family condition | Single | 54 (40-65) | 0.223 | 39 (31-46) | 0.316 |
|  | Cohabitate | 47 (37-60) |  | 35 (28-50) |  |
|  | Unclear | 55 (43-66.5) |  | 35 (30-41) |  |
| Residential condition | At home | 50.5 (39.25-63) | 0.923 | 36 (28-46) | 0.331 |
|  | Nursing home | 50 (36-72) |  | 35.5 (31.25-53.75) |  |
|  | Other | 52.5 (43.75-61.25) |  | 34.5 (28.75-36.75) |  |
| Mode of referral | EMS + EP | 48 (35-60) | 0.688 | 36 (30-54.25) | 0.617 |
|  | EMS | 53 (39.25-67.25) |  | 35 (29-45) |  |
|  | Private | 55 |  | 36 (23-60) |  |
|  | Unclear | 61 (40.5-66.5) |  | 35 (32.5-57) |  |
| EMS Pre-notification | yes | 50.5 (37.75-63.25) | 0.559 | 35 (29-45) | 0.029 |
|  | no | 49 (40-62.5) |  | 41 (34.5-60) |  |
|  | unclear | 63 (45.25-67.25) |  | 35 |  |
| Weekend vs. Weekday | Weekday | 51.5 (39.25-64) | 0.912 | 35 (29-45) | 0.052 |
|  | Weekend | 49 (40-63) |  | 38 (33-53) |  |
| Timepoint of admittance | working hours | 50 (39-68) | 0.906 | 35 (28.5-46.25) | 0.633 |
|  | on-call hours | 53 (44-60.75) |  | 36 (30-46) |  |
| No. of neurologists working in the ER | 1 | 55 (41.5-62.5) | 0.606 | 38 (31.25-52) | 0.014 |
|  | 2 | 48.5 (38.75-64.25) |  | 33 (28-43) |  |
| No. of neurological patients referred to ER within ± 1h of the referral of a stroke patient | 0 | 50.5 (42.25-58.75) | 0.908 | 36.5 (30-49.5) | 0.573 |
|  | 1-3 | 50.5 (38.25-65) |  | 35 (29-43.5) |  |
|  | 4-7 | 56 (35-75) |  | 35.5 (29.25-53.5) |  |
| Work experience of ER neurologist in charge in years | 1-3 | 51 (40-63) | 0.966 | 27 (33-45.25) | 0.013 |
|  | 4-5 | 53 (36-72) |  | 39.5 (33-54.25) |  |
|  | 6-7 | 45.5 (40-68.5) |  | 39 (30-44.5) |  |
| Acute treatment of elevated blood pressure | yes | 55 (43-65) | 0.228 | 35 (29-45) | 0.01 |
|  | no | 50 (38-63) |  | 39.5 (33.75-54) |  |
| Agitation and vomiting | yes | 53 (42-60) | 0.768 | 56 (40.25-85.25 | 0.001 |
|  | no | 50 (39.25-64.75) |  | 35 (29-45) |  |
| Consultation of relatives | yes | 54.5 (49-75.75) | 0.33 | - | 0.791 |
|  | no | 50 (39.5-63.5) |  | 36 (30-46) |  |
| Arrival without IV line | yes | 59 (42.5-71.5) | 0.168 | 41 (36-55) | 0.005 |
|  | no | 50 (39-63) |  | 35 (29-45.75) |  |
| Waiting for brain imaging | yes | 53 (41.75-62.75) | 0.489 | 51.5 (36.25-59.25) | <0.001 |
|  | no | 50 (38-64) |  | 35 (29-44) |  |
| ER treatment over 10 min with-out another reason of delay | yes | 58 (39.5-79) | 0.497 | 54.5 (36.25-64.75) | <0.001 |
|  | no | 50.5 (39.75-63.25) |  | 35 (29-44) |  |
| Delay with unclear reason | yes | 47.5 (41.75-59.75) | 0.869 | 60.5 | 0.370 |
|  | no | 51 (39-64) |  | 36 (30-46) |  |
| Indication for IVT is not clear | yes | 55 (43-65) | 0.148 | 51.5 (40-64) | <0.001 |
|  | no | 49 (38.75-60.5) |  | 34 (28.5-42.5) |  |
| Technical difficulties with rt-PA | yes | 58 | 0.874 | 33 | 0.629 |
|  | no | 50.5 (40-64) |  | 36 (30-46) |  |

Abbreviations: PHI: private health insurance, SHI: statutory health insurance, EMS: Emergency medical service, EP: emergency physician, ER: emergency room, IV: intravenous, IVT intravenous thrombolysis, rt-PA: recombinant tissue type plasminogen activator

**Supplemental Table 2:** Impact of DNT influencing factors in a univariate analysis of study phase I and II

|  | | Pre-SOP I | | Post-SOP I | | Pre-SOP II | | Post-SOP II | |
| --- | --- | --- | --- | --- | --- | --- | --- | --- | --- |
|  |  | DNT (median in min (25^th^ – 75^th^ percentile)) | p - value | DNT (median in min (25^th^ – 75^th^ percentile)) | p - value | DNT (median in min (25^th^ – 75^th^ percentile)) | p - value | DNT (median in min (25^th^ – 75^th^ percentile)) | p - value |
|  |  |  |  |  |  |  |  |  |  |
| Sex | Male | 46 (36-60) | 0.01 | 35 (29.75-44) | 0.230 | 35.5 (25-48) | 0.58 | 28 (18-40.5) | 0.577 |
|  | Female | 55 (43.25-71.5) |  | 36 (30-54) |  | 33 (27-46) |  | 30 (17-47) |  |
| Age | < 75 | 49 (37-60) | 0.252 | 33 (26-44.5) | 0.013 | 35.5 (24.25-47.25) | 0.783 | 34 (18.75-49.25) | 0.094 |
|  | ≥ 75 | 51 (40-68) |  | 37 (31-51.5) |  | 34 (27-48) |  | 26 (16-42) |  |
| NIHSS | 0-4 | 54 (39-65) | 0.367 | 35 (26-45) | 0.669 | 41 (28-56.25) | 0.012 | 35 (18.25-50) | 0.151 |
|  | 5-15 | 51 (40-64) |  | 36 (30-47.5) |  | 34 (26.5-46.25) |  | 28 (18-42) |  |
|  | 16-42 | 42 (37-53) |  | 35 (30-47.25) |  | 27 (18.5-36) |  | 22 (15-34.5) |  |
| Onset | clear | 48 (38-63) | 0.035 | 34.5 (27.75-40.25) | 0.002 | 31.5 (22-45.25) | 0.021 | 23 (16-39.5) | 0.001 |
|  | unkown | 58 (45.5-67.25) |  | 41 (30-54) |  | 38 (29.25-50) |  | 37.5 (29.25-50) |  |
| Localization of infarction | AC | 48 (38-63) | 0.006 | 35 (29-46) | 0.175 | 32.5 (22.75-44.5) | 0.014 | 26 (17-42) | 0.009 |
|  | PC | 59.5 (54.75-70) |  | 41.5 (33-47.75) |  | 43 (29.5-57.5) |  | 41.5 (27.25-54.25) |  |
| Imaging modality | CT | 46 (37-59) | <0.001 | 34 (28.25-44) | <0.001 | 30 (22-41.5) | <0.001 | 22 (16-33.5) | <0.001 |
|  | MRI | 62 (55-75) |  | 44 (38-55.5) |  | 48 (37.75-60) |  | 50 (39.75-58.25) |  |

Abbreviations: AC: anterior circulation, PC: posterior circulation, NIHSS: National Institutes of Health Stroke Scale

**Supplemental Table 3:** Number of delays per patient and median DNT per number of delays in pre- and post SOP I

|  | Pre-SOP I | | Post-SOP I | |
| --- | --- | --- | --- | --- |
| Number of delays per patient | Prevalence, n = 115 (%) | DNT (median in min (25^th^ – 75^th^ percentile)) | Prevalence, n =177 (%) | DNT (median in min (25^th^ – 75^th^ percentile)) |
| 0 | 11 (9.6) | 30 (24-35) | 55 (31.1) | 30 (23-35) |
| 1 | 67 (58.3) | 50 (40-65) | 58 (32.8) | 33 (28.75-44.25) |
| 2 | 29 (25.2) | 55 (43-64.5) | 41 (23.2) | 41 (36-51.5) |
| 3 | 7 (6.1) | 58 (54-62) | 17 (9.6) | 55 (42.5-68) |
| 4 | 1 (0.9) | 68 | 4 (2.3) | 55 (54-80) |
| 5 | - | - | 2 (1.1) | 73 |
